# Prostatic Massage

**Published:** 1905-05

**Authors:** W. L. Champion

**Affiliations:** Atlanta, Ga.; Genito-Urinary Surgery, The Halcyon


### PROSTATIC MASSAGE.*

BY W. L. CHAMPION, M.D.,
ATLANTA, GA.
Genito-Urinary Surgery, The Halcyon.

In a few words I desire to bring before this Association a comparatively recent adjunct in the treatment of deep-seated gonorrheal infection, that is indispensable in many cases, if a cure is perfected. This advancement is not original, but one that sufficient attention has not been called to, to lessen the number of infections, and gain the results in practice we all hope for. The profession as a whole will never be enlightened as to the serious nature of gonorrheal infection, and become so familiar with the disease that each and every member can knowingly inform a patient when he is no longer infected, or can safely marry. Still the work that has been accomplished within the past few years along this line—showing the large percentage of innocent women with acute pelvic troubles, due to the gonococcus, has opened the eyes of many, and it is to be hoped that in the near future, the men in our ranks, as well as laity, will not look lightly upon a disease that causes more suffering than tuberculosis or syphilis. A statement of this kind may be looked upon as a joke, or flippantly dismissed, but any gynecologist of experience, or genito-urinary surgeon, will bear out the assertion.

The careless manner in which gonorrhea is treated and dismissed by many practitioners is a stumbling-block to more rapid advancement in the treatment, and in a measure to the suppression of this disease. When the profession looks upon gonorrhea as a disseminator of infection, and endeavors to suppress the disease and limit its number of victims, as we would diphtheria, or small-

* Read before the Medical Association of Georgia, April, 1905.

pox, then we will be on a sound basis to accomplish a great deal more than is being done at present. The large number of men with deep-seated gonorrheal infection who are considered well, or told they are as well as they will ever be, not knowing that dangerous elements lie hidden or dormant in the prostate, prompted the foregoing remarks.

"The prostate gland is a sexual organ, partly glandular, partly muscular, lying in front of the bladder about the prostatic urethra. The prostate is the sexual heart. It has nothing to do with urination, and is quite passive during that act. But towards the sexual function it acts as a muscle, a sensory organ, and a gland.' This gland when involved by extension of urethral infection is relievable by prostatic massage properly applied. This does not apply to prostatic hypertrophy of old age. The relation of the prostate to the urethra makes the former more susceptible to infection than other adjacent structures. As a rule in patients that are told they have a gonorrheal cystitis, the prostate is the seat of the trouble. Many of the stubborn cases of gonorrhea, with profuse discharge, and those with only "clap shreds" in the urine, which will not respond to injections, irrigations, and the use of the sound, remain uncured because the prostate is involved, and the proper measures to give relief have not been instituted. One of the best friends and most frequent visitors to the doctor is the patient with chronic gonorrhea of years' standing, who will go for months apparenttly well, and a night out with a friend, and a few bottles of beer, produce a return of his old trouble. This condition in a vast majority of cases is due to an infected prostate, that is not cured by injection, irrigation, or the sound, which only relieve the acute condition until the exciting cause reproduces the same results. The presence of a urethral stricture of large calibre, or the endoscope showing a granular urethra does not necessarily mean that the relief of this condition perfects a cure. When the stricture has been cut, and the endoscope shows the urethra no longer granular, yet still we have pus in the urine, then we must look elsewhere for the trouble ; and usually it will be found in the prostate. Now, I am not condemning the use of injections, irrigations, or the sound, for they all have their place, but only pointing out the value of prostatic massage in deep-seated gonorrheal infection. The relief given to an extremely nervous patient, with vague pains in the pelvis, back and limbs, with a dull, heavy sensation in the rectum, after carefully massaging his sensitive and swollen prostate, is sufficient evidence of the value of the procedure. At the next visit, the first exclamation of the patient is, that you have located the trouble, and he feels greatly benefited. Also the marked improvement in the condition of the urine after the first treatment is very noticeable.

There are various positions in which patients are placed to examine and massage the prostate. I have the patients stand with elbows on seat of a chair, legs straight and feet separated. Use pressure over bladder with left hand, and massage prostate with fore-finger of right hand. The use of the rubber finger-cot makes the treatment less objectionable to the patient and more cleanly to the surgeon. The finger should be passed up to the base of the prostate, and pressure made as the finger is brought towards the apex. Firm pressure should be made over the entire gland, noting the impression made upon the patient. If he becomes faint limit the examination or massage to a few seconds and make a more thorough massage at the next treatment, when the patient will tolerate more pressure upon the gland on account of a less sensitive condition, due directly to the previous massage. In highly nervous individuals, or those in whom the gland is very sensitive, massage every fourth or fifth day is as often as advisable. In some cases I use massage only once a week, while in others, with urine cloudy, dull, heavy sensation in gland, with frequent desire to urinate, I massage every second or third day, if the patient is made more comfortable by such treatment. The amount of pressure made upon the gland, the intervals between the treatments and the length of each séance, must be determined by results obtained in each individual case. When a prostate is massaged, instruct the patient to save some urine in the bladder, so that after the treatment the urine can be voided in a glass, so as to note what secretion is compressed out of the gland. Always irrigate the bladder after massage with an antiseptic solution, so as to cleanse it of any septic material that may be present. Stones or concretions in the gland should be felt for, the condition of the vesicles and any nodules or other abnormalities noted. The vesicles are above the prostate, and lie on the outside of each vas deferens. Stripping of the vesicles is necessary to remove gonorrheal infection, and the genito-urinary surgeon is called upon to determine if the patient is sterile, by obtaining the semen to note if spermatozoa are present and alive. By massage of the prostate we compress out the infected secretion and stimulate circulation in the gland and adjacent diseased structures. Where there is a spasmodic condition of the neck of the bladder and deep urethra, massage relieves the sensitive condition and spasm, so that the patient is made more comfortable, and irrigation of the bladder is more easily accomplished. To illustrate that sensation is blunted, it is noticeable that a patient can not empty the bladder immediately after massage. Lydston has well said that "sexual neurasthenia, embracing bowel inactivity, melancholia, hypochondriasis, and that seldom recognized but frequent condition, male hysteria, are among the results of chronic prostatic disease which are frequently relieved by proper and systematic massage."

A frequently obscure condition, but one due to a local irritation, abnormal nocturnal emissions and premature ejaculation, are frequently benefited or relieved by massage. I recall a case I had a few months ago, of a boy sixteen years of age who had wet the bed every night since his birth, and a single massage caused him to pass a week without wetting the bed.

While prostatic massage is indicated in deep-seated infection due usually to gonorrhea, I mention these conditions to show the correcting influence it may have on other irregularities. This measure is not a cure-all; it is a recognized procedure that will relieve some heretofore obscure diseased conditions of the genito-urinary apparatus.

Prudential Building.

---

Any physician who has not received a recently issued panel, issued by the New York Pharmacal Association of Yonkers, graphically illustrating in colors "The Theory of Evolution" and the "Descent of Man" as promulgated by Haeckel, should write for a copy. It is interesting and decorative.

